# Correlation Between Diverse Smoking Habits and the Risk of Developing Type 2 Diabetes: A Comparative Analysis

**DOI:** 10.7759/cureus.72038

**Published:** 2024-10-21

**Authors:** Yasir Khaleel Almusawi, Fatma Azaiez, Ali B Roomi, Hajar Litaiem Ghorbel

**Affiliations:** 1 Department of Medical Laboratory Techniques, College of Health and Medical Technology, Al-Esraa University, Baghdad, IRQ; 2 Laboratory of Inorganic Chemistry, Faculty of Sciences, University of Sfax, Sfax, TUN; 3 Faculty of Pharmacy of Monastir, University of Monastir, Monastir, TUN; 4 Department of Biochemistry, College of Medicine, University of Thi-Qar, Thi-Qar, IRQ

**Keywords:** diabetes mellitus, hba1c, homa-ir, insulin resistance, smoking

## Abstract

Background: Diabetes mellitus (DM) is a prevalent metabolic illness that arises as a result of a complex interplay between genetic predisposition, environmental influences, and lifestyle choices. The precise mechanisms elucidating the association between smoking and the onset of DM remain incompletely understood, despite the proposal of several ideas. The objective of this study ‎was to conduct a comparative analysis of blood glucose levels, hemoglobin A1c (HbA1c) levels, insulin hormone levels, and homeostasis model assessment of insulin resistance (HOMA-IR) ‎levels in diabetic patients who smoked and those who did not.

Methods: The study included a total of 320 volunteers divided into four groups, with each group consisting of 80 volunteers. The first group served as the control group and consisted of ‎healthy individuals. The remaining three groups consisted of type 2 diabetes mellitus (T2DM) patients, categorized based on their smoking habits. The second ‎group comprised T2DM patients who did not smoke, the third group consisted of T2‎DM patients who smoked cigarettes, and the fourth group included T2DM patients ‎who smoked e-cigarettes.‎ At recruitment, data on age, fat %, waist circumference (cm), and body mass index (kg/m^2^) was collected. Biochemical markers measured were fasting blood glucose (FBG), HbA1c, insulin, and HOMA-IR‎ levels.

Results: The findings demonstrated a statistically significant increase (P<0.001) in the levels of each parameter, particularly among patients with T2DM who engaged in e-cigarette smoking, compared to the control group.‎ It was found that engaging in dual smoking, which involves the use of both traditional cigarettes and e-cigarettes, was associated with a higher likelihood of elevated HbA1c levels and other negative health effects.

Conclusion: When it comes to the management of diabetic patients, abstaining from smoking and participating in smoking cessation programs, for patients who smoke, should be the essential approaches. It was found that dual smoking led to a higher likelihood of elevated HbA1c levels and this association was most pronounced among male individuals, those who were physically inactive, and those classified as obese. Further studies should be conducted on the detrimental health consequences associated with e-cigarettes, with a particular focus on enhancing the awareness of healthcare professionals and their patients of the potential risks. This is particularly significant due to the prevailing perception that e-cigarettes are inherently "safe".

## Introduction

Smoking is considered a modifiable risk factor for various chronic diseases, including cardiovascular disease (CVD), cancer, chronic obstructive lung disease, asthma, and diabetes. Nevertheless, the negative effects of smoking on diabetes have largely been insufficiently acknowledged. According to the guidelines provided by the Korean Diabetes Association, the act of ceasing smoking is advised as a crucial measure in the prevention of cardiovascular complications associated with diabetes [1].

Numerous researches have demonstrated that smoking has detrimental effects on diabetes mellitus, specifically in relation to diabetic macrovascular problems. However, the precise causal relationship between smoking and the evolution of diabetic microvascular complications remains unexplored. Type 2 diabetes mellitus (T2DM) is a significant public health concern on a global scale, with a prevalence of around 1 in 11 persons in year 2015 [2]. Observational studies have suggested that cigarette smoking may serve as a distinct and adjustable risk factor for type 2 diabetes, applicable to both males and females [3,4]. The precise underlying mechanism through which smoking elevates the likelihood of developing diabetes and disrupts glucose regulation remains incompletely understood. However, the existing research indicates that smoking contributes to the development of insulin resistance. In the context of healthy young males, in the short term, the act of smoking was observed to be associated with a heightened state of insulin resistance [3]. After smoking, individuals who smoked experienced a notable elevation in their homeostatic model assessment of insulin resistance (HOMA-IR) levels after one hour [5]. In male individuals who engages in smoking, there was a notable decrease in insulin-mediated glucose absorption ranging from 10% to 40% when compared to their non-smoking counterparts [6,7]. In individuals with type 2 diabetes, the insulin and C-peptide responses to an oral glucose load were found to be notably elevated in smokers compared to non-smokers. Additionally, the degree of insulin resistance, as assessed using the euglycemic clamp technique, exhibited a positive correlation that was dependent on the dosage [8]. Therefore, smoking has been found to cause insulin resistance in individuals diagnosed with type 2 diabetes, as well as in individuals without any preexisting health conditions. Glycated hemoglobin (HbA1c) serves as a well-accepted diagnostic tool for diabetes on a global scale. The application of HbA1c as a diagnostic instrument presents some advantages when compared to a fasting blood glucose (FBG) examination. As a result, it has been recommended for the evaluation of glucose regulation in persons who have already been diagnosed with diabetes [9,10]. The HbA1c level functions as a metric for evaluating the mean blood glucose concentration over a span of approximately two to three months. Consequently, it holds significant utility in the evaluation and regulation of glycemic control in persons afflicted with diabetes [11,12].

Electronic cigarettes, also known as e-cigarettes, are devices that transform nicotine into vapour, purporting to possess reduced quantities of traditional contaminants in comparison to the emissions from the secondhand tobacco smoke. These gadgets are specifically engineered to replicate the action of consuming conventional cigarettes. These devices are widely utilized by individuals seeking to quit or decrease their cigarette consumption, as they are effective tools for smoking cessation and reduction [13-15]. Numerous research studies have documented the positive impacts of electronic cigarettes in this regard [16-17]. Nevertheless, the comprehensive understanding of the impacts of e-cigarette usage is not as well established as that of traditional cigarettes. Consequently, the safety of these devices for persons seeking to quit or reduce smoking remains unknown. Furthermore, the practice of dual smoking, which involves the concurrent use of both e-cigarettes and traditional cigarettes, has been found to potentially contribute to tobacco addiction, but its additional impacts on health remain uncertain [18].

In contrast to the ample information available on the association between cigarette smoking and many health outcomes, the current body of research falls short in providing adequate evidence to elucidate the relationship between dual smoking or e-cigarette smoking and blood glucose levels, HbA1c levels, insulin resistance, and HOMA-IR levels. Hence, this research endeavor aimed to examine the correlation of diverse smoking habits, including dual smoking, exclusive cigarette use, and past smoking, with all the aforementioned parameters within a representative sample of the community.

## Materials and methods

Data collection

This cross-sectional study was based on the data collected from February 2022 to January 2023, from patients and volunteers at the Baghdad General Hospital, Baghdad; it was approved by the ethical committee of the hospital (IRB no. 2/2022). The study had a total of 320 male‎ volunteers, divided into four groups, with each group consisting of 80 volunteers. The first group served as the control group and consisted of ‎healthy individuals. The remaining three groups consisted of T2DM patients, categorized based on their smoking habits. The second ‎group comprised T2DM patients who did not smoke, the third group consisted of T2DM patients who smoked cigarettes, and the fourth group included T2DM patients ‎who smoked e-cigarettes.‎ Every participant gave their informed consent for participating in the present study. The following equation was used to determine the sample size required for our study: ‎n = Z^2 ^* P (1-P)/d^2^, where n is the sample size, Z is the Z statistic for a level of confidence, P is the expected ‎prevalence or proportion and d is the precision (in proportion of one, if 5%, d ‎‎= 0.01).‎ Participants with a liver disease, renal disease, endocrine or inflammatory disorders, taking other medications, or with irregularities in their diet or exercise routine were excluded. Information on patients' age, fat %, waist circumference (cm) and BMI (kg/m^2^) was collected for each group. All samples were centrifuged at 3000 rpm for five minutes, and several parameters were measured.

Fasting Blood Sugar

To measure this, 1 ml of the reagent was added to 1 µl of the serum incubated for five minutes in a water bath at 37°C. The absorbance of the sample was recorded at 500 nm and the concentration of glucose was calculated using the following formula: Glucose (mg/dl) = (A sample)/(A standard) * standard concentration (100).

HbA1c Level

The Finecare™ HbA1c Rapid Quantitative Test (Wondfo Biotech, Guangzhou, China) using fluorescence immunoassay technology was employed, which utilizes a sandwich immunodetection method. In this procedure, the introduction of a sample into the designated sample of the test cartridge results in the binding of fluorescence-labeled detector HbA1c antibodies and detector Hb antibodies, located on the sample pad, to HbA1c antigens and Hb antigens found inside the blood specimen, respectively. The aforementioned process of binding leads to the creation of immunological complexes. Following the manual instructions, 10 µl of whole blood was added to the detection buffer tube using a transfer pipette. The cap of the buffer tube was securely fastened, and the sample mixture was well mixed by stirring it for a duration of one minute. Then, 75 μl of the sample mixture was loaded onto the sample of the test cartridge. The experiment was carried out at a room temperature of 37°C.

Serum Insulin Hormone Level

All reagents, working standards, and samples were prepared. First, 25 μl was added to each well's sample and standard control. Then 25 μl of the enzyme conjugate was added to each well. It was mixed well for a few seconds and then placed in a container for 30 minutes at room temperature.

The contents of the wells were stirred. The wells were rinsed more than twice with a diluted washing solution (400 μl per well), and allowed to dry completely. Then, 50 μl of the enzyme complex was added to each well and into the incubator for 30 minutes at room temperature. Once again, the contents of the wells were stirred. The wells were rinsed multiple times using a diluted washing solution at a volume of 400 μl per well. The wells were gently tapped with the absorbent paper in order to eliminate any remaining drops. Subsequently, a volume of 50 μl of the substrate solution was introduced into every individual well. The sample was subjected to incubation for a duration of 15 minutes at ambient temperature. A volume of 50 μl of the stop solution was introduced into each well in order to terminate the reaction. The optical density (OD) of each well was measured using a microplate reader set to a wavelength of 450 nm, and this measurement was completed within a time frame of 10 minutes. The concentration of samples corresponding to the average absorbance was calculated from the standard curve.

HOMA-IR

We used the blood glucose measurements as well as insulin measured from fasting blood glucose samples in order to calculate the IR by HOMA-IR using the formula, HOMA-IR = Glucose (mmol/l) x Insulin (µIU/ml). We considered patients insulin-resistant when HOMA-IR ≥ 3.6 and non-insulin-resistant when HOMA-IR <3.6.

Statistical analysis

Results were statistically analyzed using IBM SPSS Statistics, version 24.0 (IBM Corp., Armonk, NY). Means and standard deviations were used to express values, and one-way ANOVA was used to evaluate group differences; p<0.001 was the threshold used for determining the significant difference.

## Results

As can be seen in Table 1, all groups had approximately same results in relation to age, fat %, waist circumference (cm), and BMI (kg/m^2^).

**Table 1:** Data on age, fat %, waist circumference, and BMI for each group T2D, type 2 diabetes; ns, not significant

Parameters	Control group, mean ± SD (n=80)‎	T2D, mean ± SD (n=80)	T2D with cigarettes, ‎mean ± SD (n=80)	T2D with e-‎cigarettes, mean ± SD ‎(n=80)‎	P-value
Age (years)	30.24 ± 3.14	30.41 ± 3.92	30.56 ± 3.45	‎30.84 ± 3.25	0.172^ns^
Fat %	‎8.54 ± 1.52	8.32 ± 1.41	8.63 ± 1.12	‎8.75 ± 1.63‎	0.169^ns^
Waist circumference (cm)	‎65.25 ± 1.54	65.41 ± 1.44	65.12 ± 1.32	‎65.55 ± 1.52‎	0.152^ns^
BMI (kg/m^2^)	25.12 ± 1.78	25.21 ± 1.87	25.54 ± 1.95	‎25.75 ± 1.98	ns

**Figure 1:**
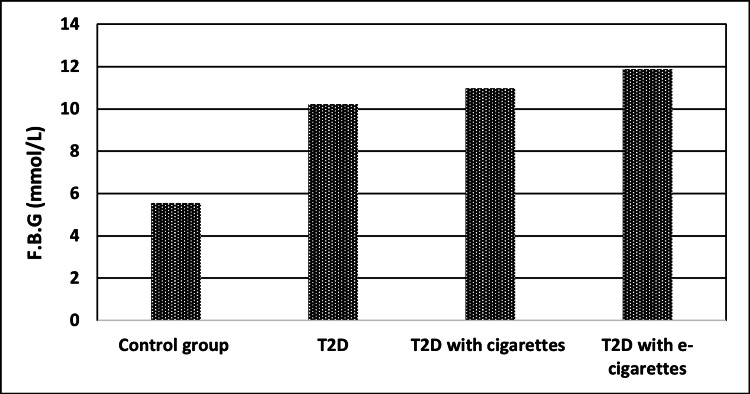
FBG (mmol/l) levels for control, T2D, T2D with cigarettes, and T2D with e-cigarette groups FBG, fasting blood glucose; T2D, type 2 diabetes

The first parameter FBG showed the difference between each group in comparison to the control group, at P<0.001, indicating that this result was more significant. The T2D with cigarette group had a higher FBG concentration (10.98 ± 1.25 mmol/l) than other groups, and the T2D with e-cigarette group had a higher concentration (11.87% ± 1.52 mmol/l) than all other groups when compared to the control group, which was 5.55 ± 1.21 mmol/l. This indicated that e-cigarettes are more dangerous for patients with T2D, as indicated in Table 2 and Figure 1.

**Table 2:** Fasting blood glucose results for control, T2D, T2D with cigarette, T2D with e-cigarette groups T2D, type 2 diabetes *P-value indicates the statistically significant difference between the control group and the T2D patient groups, including subgroups (T2D with cigarettes and T2D with e-cigarettes).

Study group	Fasting blood glucose (mmol/l)	P-value
Control group, mean ± SD (n=80)‎	5.55 ± 1.21	<0.001*
T2D, mean ± SD (n=80)	10.21 ± 1.52	
T2D with cigarettes, mean ± SD (n=80)	10.98 ± 1.25	
T2D with e-‎cigarettes, ‎mean ± SD (n=80)‎	‎11.87 ± 1.52	

**Figure 2:**
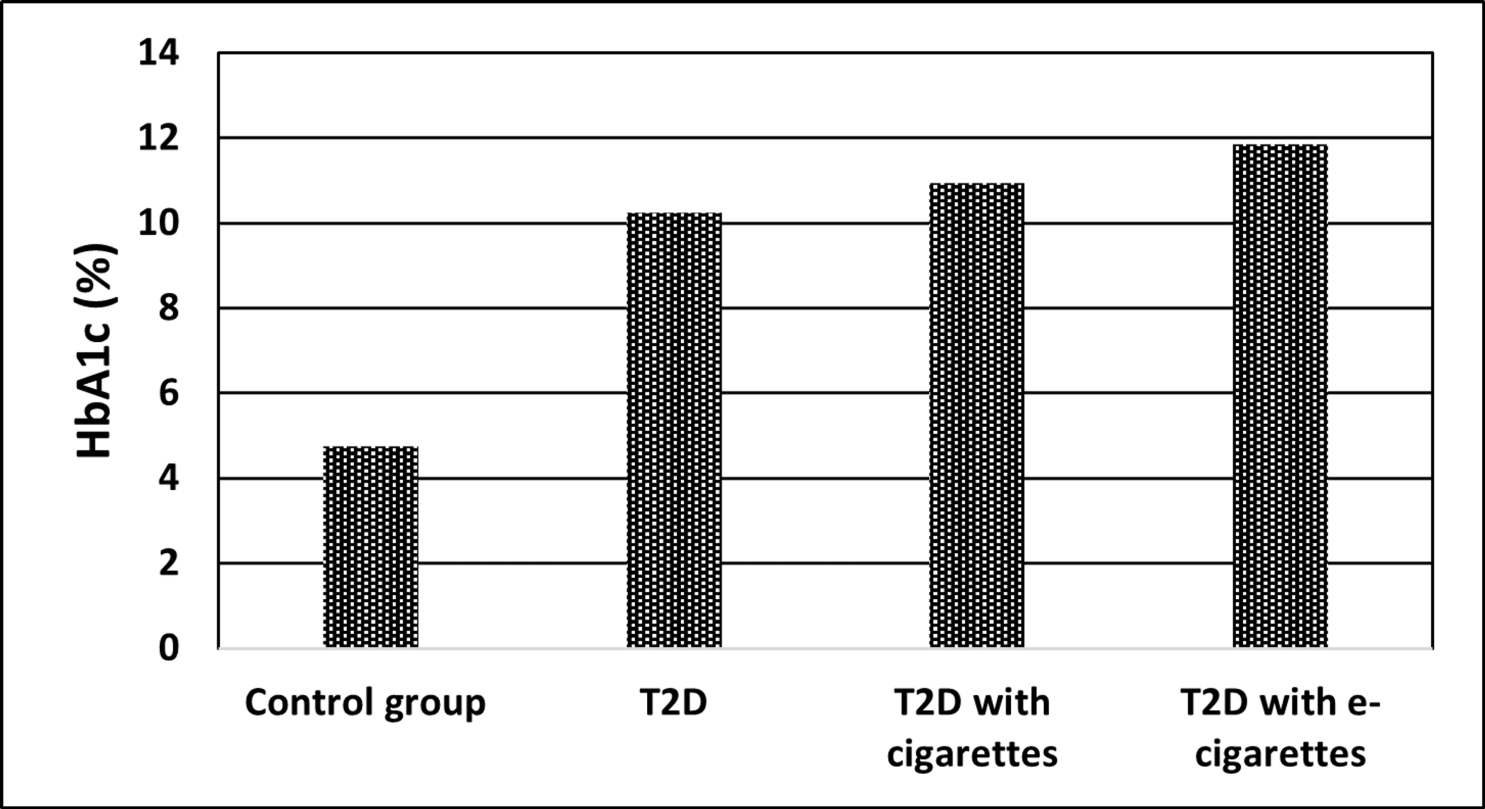
HbA1c (%) levels for control, T2D, T2D with cigarette, and T2D with e-cigarette groups T2D, type 2 diabetes

Regarding HbA1c results, the group with T2D with cigarettes and the group with T2D with e-cigarettes had significantly higher HbA1c levels than healthy patients (control, 4.74 ± 1.12%; T2D with cigarettes, 10.95 ± 1.45%; T2D with e-cigarettes, 11.84 ± 1.47%; P < 0.001) as shown in Table 3 and Figure 2.

**Table 3:** HbA1c (%) results for control, T2D, T2D with cigarette, and T2D with e-cigarette groups T2D, type 2 diabetes *P-value indicates the statistically significant difference between the control group and the T2D patient groups, including subgroups (T2D with cigarettes and T2D with e-cigarettes).

Study group	HbA1c (%)	P-value
Control group, mean ± SD (n=80)‎	4.74 ± 1.12	<0.001*
T2D, mean ± SD (n=80)	10.24 ± 1.59	
T2D with cigarettes, ‎mean ± SD (n=80)	10.95 ± 1.45	
T2D with e-‎cigarettes, ‎mean ± SD (n=80)‎	‎11.84 ± 1.47	

**Figure 3:**
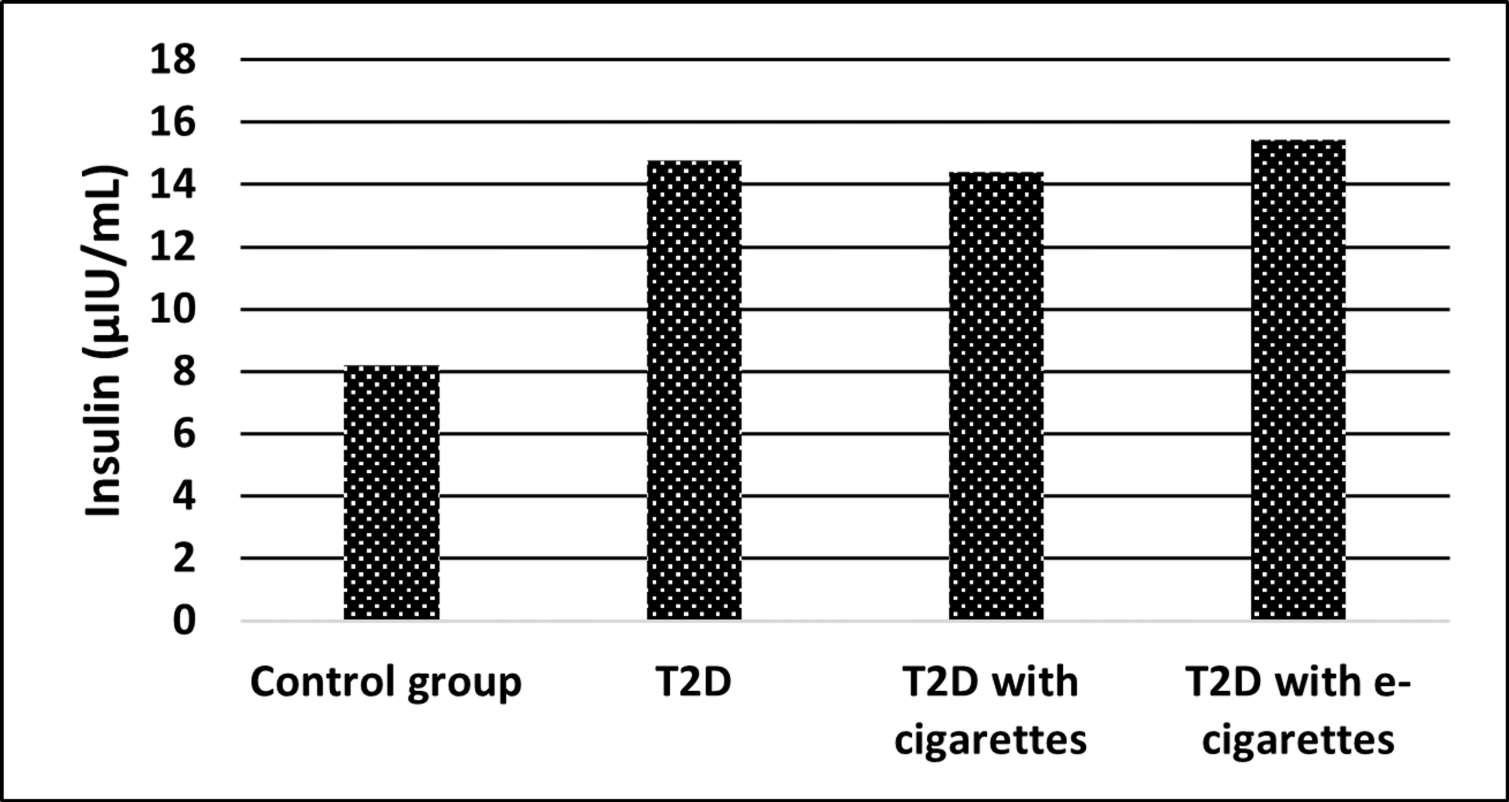
Insulin levels for control, T2D, T2D with cigarette, and T2D with e-cigarette groups T2D, type 2 diabetes

Insulin levels were significantly increased in the T2D with cigarette group compared with the control group (control, 8.21 ± 1.46 µIU/ml; T2D with cigarettes, 14.41 ± 1.74 µIU/ml) and in the T2D with e-cigarette smoking group when compared with the control group (control, 8.21 ± 1.46 µIU/ml; T2D with e-cigarettes, 15.45 ± 1.14 µIU/ml) as shown in Table 4 and Figure 3.

**Table 4:** Insulin results for control, T2D, T2D with cigarette, and T2D with e-cigarette groups T2D, type 2 diabetes *P-value indicates the statistically significant difference between the control group and the T2D patient groups, including subgroups (T2D with cigarettes and T2D with e-cigarettes).

Study group	Insulin (µIU/ml)‎	P-value
Control group, mean ± SD (n=80)‎	8.21 ± 1.46	<0.001*
T2D, mean ± SD (n=80)	14.78 ± 1.41	
T2D with cigarettes, ‎mean ± SD (n=80)	14.41 ± 1.74	
T2D with e-‎cigarettes,‎ mean ± SD (n=80)‎	‎15.45 ± 1.14‎	

**Figure 4:**
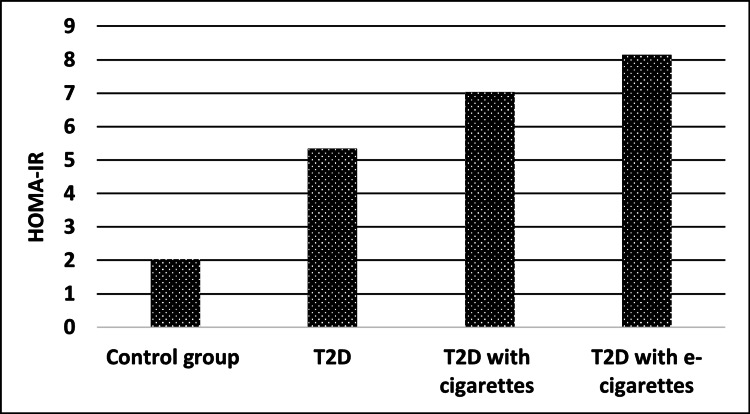
HOMA-IR levels for control, T2D, T2D with cigarette, and T2D with e-cigarette groups T2D, type 2 diabetes; HOMA-IR, homeostasis model assessment of insulin resistance

HOMA-IR levels for T2D in the cigarette group and T2D in the e-cigarette group were each significantly higher among cases compared with the control group (control, ‎‎2.02‎‏ ‏‎± ‎‏0‏‎.94; T2D with cigarettes, 7.03 ‎‏± ‏‎0‎‏.‏‎81‎‏; T‏2‏D with e-cigarettes, ‏‎8‎‏.‏‎15‎‏± ‏ ‎0‎‏.‏‎89‎‏)‏. HOMA-IR was more strongly associated with an increased risk than were other ‏markers after we excluded those with a fasting glucose level of ≥126 mg/dl at baseline, as shown in Table 5 and Figure 4.‎

**Table 5:** HOMA-IR results for control, T2D, T2D with cigarette, and T2D with e-cigarette groups T2D, type 2 diabetes; HOMA-IR, homeostasis model assessment of insulin resistance *P-value indicates the statistically significant difference between the control group and the T2D patient groups, including subgroups (T2D with cigarettes and T2D with e-cigarettes).

Study group	HOMA-IR	P-value
Control group, mean ± SD (n=80)‎	2.02‎ ± 0.94	<0.001*
T2D, mean ± SD (n=80)	5.34 ‎± 0.89‎	
T2D with cigarettes, ‎mean ± SD (n=80)	7.03 ‎± 0.81	
T2D with e-‎cigarettes,‎ mean ± SD (n=80)‎	‎8.15 ‎± 0.89	

## Discussion

There exists substantial evidence supporting the notion that smoking is associated with an elevated risk of developing diabetes. Multiple cohort studies conducted in Korea have documented a positive correlation between smoking behavior and an elevated susceptibility to the onset of diabetes [18,19]. A study was conducted in Korea, where a cohort of 4,041 men was observed over a period of four years in both rural and urban areas [18]. The study revealed a noteworthy association between smoking habits and the risk of developing type 2 diabetes. Specifically, individuals who had a history of smoking as well as those who were currently smoking exhibited a significantly higher risk for type 2 diabetes. Furthermore, the magnitude of this risk was found to escalate in proportion to the number of cigarettes consumed.

Recent studies demonstrated a positive correlation between cigarette smoking and elevated levels of HbA1c in individuals who did not have diabetes. A prior investigation demonstrated a positive correlation between cigarette smoking and elevated levels of HbA1c within the broader population [20,21]. Furthermore, a study revealed that individuals who use e-cigarettes exhibit a higher level of HbA1c in comparison to those who use traditional cigarettes [22].

The adverse impact of cigarette smoking on insulin sensitivity has been well demonstrated. There have been reports of epidemiological evidence indicating a correlation between smoking and the development of insulin resistance [21-23]. Eliasson et al. discovered a significant correlation between the daily consumption of cigarettes and the level of insulin resistance [5,22]. In a study, it was found that smokers had a high risk of developing insulin resistance compared with a matched group of non-smokers [24].

Our study also has certain limitations. Even though there is a lot of evidence linking smoking (cigarettes‎ and e-cigarettes‎) ‎to a higher risk of developing diabetes, a careful interpretation of results is needed. Further studies are required to identify the underlying mechanisms and establish causal linkages.

## Conclusions

This study revealed that smoking habits, particularly of those who engage in dual smoking by using both e-cigarettes and traditional cigarettes, are associated with a higher likelihood of elevated HbA1c levels in the general population. This association is most pronounced among male individuals, those who are physically inactive, and those classified as obese. This study is an early attempt to investigate the collective and separate impacts of conventional cigarettes and electronic cigarettes on HbA1c levels. Therefore, it is crucial to provide patients with education regarding the significance of abstaining from smoking and participating in smoking cessation programs as essential approaches for the management of diabetes. Although engaging in dual smoking has been associated with negative health effects, the impact of e-cigarette use on other health outcomes still lacks clarity. Hence, it is imperative to conduct further investigations into the detrimental health consequences associated with e-cigarettes, with a particular focus on enhancing the awareness of healthcare professionals and their patients of the potential risks posed by these devices. This aspect assumes particular significance due to the prevailing perception among a substantial number of individuals that e-cigarettes are inherently "safe".
